# Genome sequence of the *Lotus spp.* microsymbiont *Mesorhizobium loti* strain NZP2037

**DOI:** 10.1186/1944-3277-9-7

**Published:** 2014-12-08

**Authors:** Simon Kelly, John Sullivan, Clive Ronson, Rui Tian, Lambert Bräu, Karen Davenport, Hajnalka Daligault, Tracy Erkkila, Lynne Goodwin, Wei Gu, Christine Munk, Hazuki Teshima, Yan Xu, Patrick Chain, Tanja Woyke, Konstantinos Liolios, Amrita Pati, Konstantinos Mavromatis, Victor Markowitz, Natalia Ivanova, Nikos Kyrpides, Wayne Reeve

**Affiliations:** 1 Department of Microbiology and Immunology, University of Otago, Dunedin, New Zealand; 2 Centre for Rhizobium Studies, Murdoch University, Murdoch, Perth, Australia; 3 School of Life and Environmental Sciences, Deakin University, Deakin, Victoria, Australia; 4 Los Alamos National Laboratory, Bioscience Division, Los Alamos, NM, USA; 5 DOE Joint Genome Institute, Walnut Creek, CA, USA; 6 Biological Data Management and Technology Center, Lawrence Berkeley National Laboratory, Berkeley, CA, USA; 7 Department of Biological Sciences, King Abdulaziz University, Jeddah, Saudi Arabia

**Keywords:** Root-nodule bacteria, Nitrogen fixation, Symbiosis, *Alphaproteobacteria*

## Abstract

*Mesorhizobium loti* strain NZP2037 was isolated in 1961 in Palmerston North, New Zealand from a *Lotus divaricatus* root nodule. Compared to most other *M. loti* strains, it has a broad host range and is one of very few *M. loti* strains able to form effective nodules on the agriculturally important legume *Lotus pedunculatus*. NZP2037 is an aerobic, Gram negative, non-spore-forming rod. This report reveals that the genome of *M. loti* strain NZP2037 does not harbor any plasmids and contains a single scaffold of size 7,462,792 bp which encodes 7,318 protein-coding genes and 70 RNA-only encoding genes. This rhizobial genome is one of 100 sequenced as part of the DOE Joint Genome Institute 2010 *Genomic Encyclopedia for Bacteria and Archaea-Root Nodule Bacteria* (GEBA-RNB) project.

## Introduction

*Mesorhizobium loti* strain NZP2037 (ICMP1326) was isolated in 1961 from a root nodule off a *Lotus divaricatus* plant growing near Palmerston North airport, New Zealand [1]. Strain NZP2037 is distinguished from most other strains of *M. loti* by its broad host range (see below), including the ability to form effective nodules on the agriculturally important legume *Lotus pedunculatus* (syn. *L. uliginosus*) [2]. Most *M. loti* strains, including the type strain NZP2213, are only able to induce uninfected nodule primordia on this host [2,3].

The ability of *M. loti* strains to form effective nodules on *L. pedunculatus* was correlated with their ‘*in vitro*’ sensitivity to flavolans (condensed tannins) present in high concentration in the roots of this legume [4]. The resistance of *M. loti* strain NZP2037 to flavolans from *L. pedunculatus* was associated with the presence of a strain-specific polysaccharide component in the outer cell membrane complex of the bacterium [5]. However the genes required for the synthesis of this flavolan-binding polysaccharide have not been identified and whether the polysaccharide is necessary for nodulation of *L. pedunculatus* has not been established.

Nodulation and nitrogen fixation genes in *Mesorhizobium loti* strains are encoded on the chromosome on acquired genetic elements termed symbiosis islands [6]. The sequence of the strain NZP2037 symbiosis island was recently reported and it was found that it was split into two regions of 528 kb and 5 kb as the result of a large-scale genome rearrangement [7]. This observation is confirmed by the whole-genome sequence reported in this paper. The Nod factor produced by NZP2037 contains an extra carbamoyl group at its non-reducing end compared to that produced by most other *M. loti* strains [8] and the NZP2037 symbiosis island contains a *nodU* gene that is likely responsible for this modification [7]. The symbiosis island was also found to contain *nodFEGA* genes absent from *M. loti* strain R7A that may lead to the incorporation of unsaturated fatty acid moieties on the Nod factor [7]. Whether these genes contribute to the broad host range of strain NZP2037 has not been reported.

The broad host range of NZP2037 was exploited by Hotter and Scott [9] to show that rhizobial exopolysaccharide was required for the formation of infected nodules on the indeterminate host *Leucaena leucocephala* but not on the determinate nodulating host *L. pedunculatus*. This observation supported suggestions that acidic EPS is required for effective nodulation of indeterminate but not determinate nodulating legumes (reviewed by [10]). However recent work by Kelly *et al*. using *M. loti* strain R7A showed that certain rhizobial exopolysaccharide mutants including *exoU* mutants induced only uninfected nodules on *L. corniculatus*, supporting a role for exopolysaccharide in determinate nodulation [11]. Interestingly, *exoU* mutants of NZP2037 form effective nodules on *L. corniculatus*[12], again suggesting that NZP2037 may produce a strain-specific surface polysaccharide that plays a symbiotic role.

Here we present a summary classification and a set of general features for *M. loti* strain NZP2037 together with the description of the complete genome sequence and annotation.

### Classification and general features

**Figure 1:**
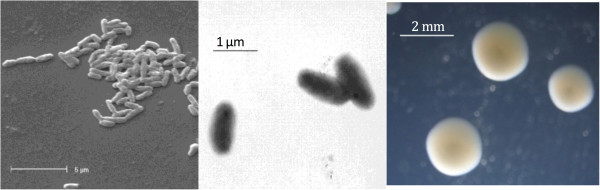
**Images of *Mesorhizobium loti *** **strain NZP2037 using scanning (left) and transmission (center) electron microscopy and the appearance of colony morphology on ½LA (right).**

*Mesorhizobium loti* strain NZP2037 is in the order *Rhizobiales* of the class *Alphaproteobacteria*. Cells are described as non-sporulating, Gram-negative, non-encapsulated, rods. The rod-shaped form varies in size with dimensions of 0.5-0.75 μm in width and 1.25-1.5 μm in length (Figure 1 left and center). They are moderately fast growing, forming 2 mm diameter colonies within 5 days and have a mean generation time of approximately 6 h when grown in TY broth at 28°C [13]. Colonies on G/RDM agar [14] and half strength Lupin Agar (½LA) [15] are opaque, slightly domed, mucoid with smooth margins (Figure 1 right).

Strains of this organism are able to tolerate a pH range between 4 and 10. Carbon source utilization and fatty acid profiles of *M. loti* have been described previously [3,16,17]. Minimum Information about the Genome Sequence (MIGS) is provided in Table 1.

**Table 1:** **Classification and general features of *Mesorhizobium loti *** **strain NZP2037 according to the MIGS recommendations**[18,19]

**MIGS ID**	**Property**	**Term**	**Evidence code**
	Current classification	Domain *Bacteria*	TAS [19]
		Phylum *Proteobacteria*	TAS [20]
		Class *Alphaproteobacteria*	TAS [21]
		Order *Rhizobiales*	TAS [22,23]
		Family *Phyllobacteriaceae*	TAS [23,24]
		Genus *Mesorhizobium*	TAS [16]
		Species *Mesorhizobium loti*	TAS [3]
		Strain NZP2037	TAS [3]
	Gram stain	Negative	IDA
	Cell shape	Rod	IDA
	Motility	Motile	IDA
	Sporulation	Non-sporulating	NAS
	Temperature range	Mesophile	NAS
	Optimum temperature	28°C	NAS
	Salinity	Unknown	NAS
MIGS-22	Oxygen requirement	Aerobic	TAS [3]
	Carbon source	Various	TAS [16,25]
	Energy source	chemoorganotroph	TAS [16,25]
MIGS-6	Habitat	Soil, root nodule, host	TAS [3]
MIGS-15	Biotic relationship	Free living, Symbiotic	TAS [3]
MIGS-14	Pathogenicity	None	NAS
	Biosafety level	1	TAS [26]
	Isolation	Root nodule of *Lotus divaricatus*	TAS [27]
MIGS-4	Geographic location	Adjacent Palmerston North Airport, NZ	TAS [1]
MIGS-5	Nodule collection date	1961	TAS [1]
MIGS-4.1	Latitude	-40.1914	TAS [1]
MIGS-4.2	Longitude	175.3701	TAS [1]
MIGS-4.3	Depth	5 cm	IDA
MIGS-4.4	Altitude	46 meters	IDA

Evidence codes – IDA: Inferred from Direct Assay; TAS: Traceable Author Statement (i.e., a direct report exists in the literature); NAS: Non-traceable Author Statement (i.e., not directly observed for the living, isolated sample, but based on a generally accepted property for the species, or anecdotal evidence). These evidence codes are from the Gene Ontology project [28].

**Figure 2:**
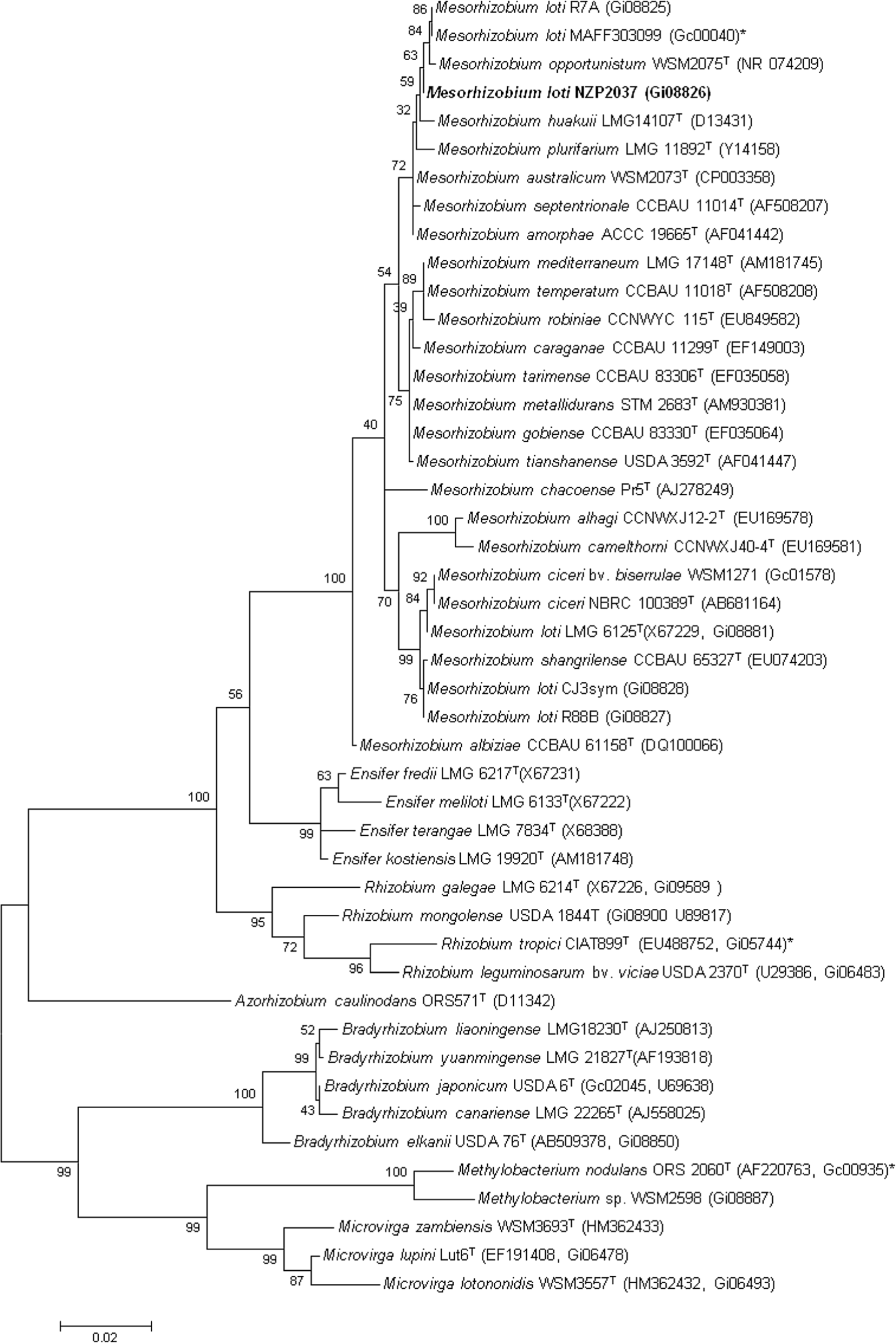
**Phylogenetic tree showing the relationships of *Mesorhizobium. loti *NZP2037 with other root nodule bacteria based on aligned sequences of the 16S rRNA gene (1,290 bp internal region).** All sites were informative and there were no gap-containing sites. Phylogenetic analyses were performed using MEGA [29], version 5. The tree was built using the Maximum-Likelihood method with the General Time Reversible model [30]. Bootstrap analysis [31] with 500 replicates was performed to assess the support of the clusters. Type strains are indicated with a superscript T. Brackets after the strain name contain a DNA database accession number and/or a GOLD ID (beginning with the prefix G) for a sequencing project registered in GOLD [32]. Published genomes are indicated with an asterisk.

Figure 2 shows the phylogenetic neighborhood of *M. loti* strain NZP2037 in a 16S rRNA gene sequence based tree. This strain has 99.7% (1,363/1,367 bp) 16S rRNA gene sequence identity to *M. loti* MAFF303099 (GOLD ID: Gc00040) and 99.6% sequence identity (1,362/1,397 bp) to *M. opportunistum* WSM2075 (GOLD ID: Gc01853).

### Symbiotaxonomy

Like most other *M. loti* strains including the type strain NZP2213, strain NZP2037 forms effective nodules on *Lotus corniculatus, L. tenuis, L. japonicus, L. burttii, L. krylovii, L. filicaulis* and *L. schoelleri*[2,33]. However, it also forms nitrogen-fixing nodules on several hosts that strain NZP2213 only induces uninfected nodules on. These hosts include *Lotus pedunculatus, L. angustissimus, L. subbiflorus, Leuceana leucocephala, Carmichaelia flagelliformis, Ornithopus sativus* and *Clianthus puniceus*[33].

### Genome sequencing and annotation information

#### Genome project history

This organism was selected for sequencing on the basis of its environmental and agricultural relevance to issues in global carbon cycling, alternative energy production, and biogeochemical importance, and is part of the Community Sequencing Program at the U.S. Department of Energy, Joint Genome Institute (JGI) for projects of relevance to agency missions. The genome project is deposited in the Genomes OnLine Database [32] and a high-quality-draft genome sequence in IMG. Sequencing, finishing and annotation were performed by the JGI. A summary of the project information is shown in Table 2.

**Table 2:** **Genome sequencing project information for *Mesorhizobium loti *** **NZP2037**

**MIGS ID**	**Property**	**Term**
MIGS-31	Finishing quality	High-quality-draft
MIGS-28	Libraries used	Illumina Standard (short PE) and CLIP (long PE) libraries
MIGS-29	Sequencing platforms	Illumina HiSeq2000 technology
MIGS-31.2	Sequencing coverage	Illumina: 509×
MIGS-30	Assemblers	Velvet version 1.1.05; Allpaths-LG version r39750 phrap, version 4.24
MIGS-32	Gene calling method	Prodigal 1.4, GenePRIMP
	Genbank accession	AQZP00000000
	Genbank Registration Date	September 16, 2013
	GOLD ID	Gi08826
	NCBI project ID	81803
	Database: IMG	2517572076
	Project relevance	Symbiotic nitrogen fixation, agriculture

#### Growth conditions and DNA isolation

*M. loti* strain NZP2037 was grown to mid logarithmic phase in TY rich medium [34] on a gyratory shaker at 28°C at 250 rpm. DNA was isolated from 60 mL of cells using a CTAB (Cetyl trimethyl ammonium bromide) bacterial genomic DNA isolation method [35].

### Genome sequencing and assembly

The draft genome of *M. loti* NZP2037 was generated at the DOE Joint Genome Institute (JGI) using Illumina technology [36]. For this genome, we constructed and sequenced an Illumina short-insert paired-end library with an average insert size of 270 bp which generated 9,401,642 reads and an Illumina long-insert paired-end library with an average insert size of 3047.66 +/- 2184.11 bp which generated 16,067,290 reads totaling 3,820 Mbp of Illumina data. (unpublished, Feng Chen). All general aspects of library construction and sequencing performed at the JGI can be found at the JGI website [37].

The initial draft assembly contained 13 contigs in 6 scaffolds. The initial draft data was assembled with Allpaths, version 39750, and the consensus was computationally shredded into 10 Kbp overlapping fake reads (shreds). The Illumina draft data was also assembled with Velvet [38], version 1.1.05, and the consensus sequences were computationally shredded into 1.5 Kbp overlapping fake reads (shreds). The Illumina draft data was assembled again with Velvet using the shreds from the first Velvet assembly to guide the next assembly. The consensus from the second VELVET assembly was shredded into 1.5 Kbp overlapping fake reads. The fake reads from the Allpaths assembly and both Velvet assemblies and a subset of the Illumina CLIP paired-end reads were assembled using parallel phrap, version 4.24 (High Performance Software, LLC). Possible mis-assemblies were corrected with manual editing in Consed [38-41]. Gap closure was accomplished using repeat resolution software (Wei Gu, unpublished), and sequencing of bridging PCR fragments with Sanger technology. The total ("estimated size" for unfinished) size of the genome is 7.5 Mbp and the final assembly is based on 3,820 Mbp of Illumina draft data, which provides an average 509× coverage of the genome.

#### Genome annotation

Genes were identified using Prodigal [42] as part of the DOE-JGI genome annotation pipeline, followed by a round of manual curation using the JGI GenePrimp pipeline [43]. The predicted CDSs were translated and used to search the National Center for Biotechnology Information (NCBI) nonredundant database, UniProt, TIGRFam, Pfam, PRIAM, KEGG, COG, and InterPro databases. These data sources were combined to assert a product description for each predicted protein. Non-coding genes and miscellaneous features were predicted using tRNAscan-SE [44], RNAMMer [45], Rfam [46], TMHMM [47], and SignalP [48]. Additional gene prediction analyses and functional annotation were performed within the Integrated Microbial Genomes (IMG-ER) platform [49,50].

### Genome properties

**Figure 3:**
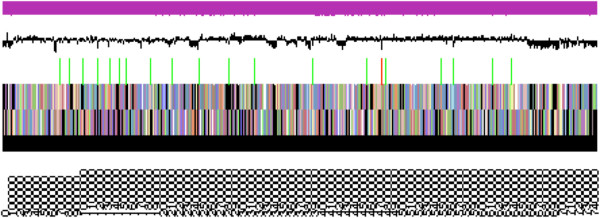
**Graphical map of the single scaffold of *Mesorhizobium loti *NZP2037.** From bottom to the top: Genes on forward strand (color by COG categories as denoted by the IMG platform), Genes on reverse strand (color by COG categories), RNA genes (tRNAs green, sRNAs red, other RNAs black), GC content, GC skew.

The genome is 7,462,792 nucleotides with 62.76% GC content (Table 3 and Figure 3) and is comprised of a single scaffold and no plasmids. From a total of 7,388 genes, 7,318 were protein encoding and 70 RNA-only encoding genes. Within the genome, 286 pseudogenes were also identified. The majority of genes (80.97%) were assigned a putative function while the remaining genes were annotated as hypothetical. The distribution of genes into COGs functional categories is presented in Table 4.

**Table 3:** **Genome statistics for *Mesorhizobium loti *** **NZP2037**

**Attribute**	**Value**	**% of total**
Genome size (bp)	7,462,792	100.00
DNA coding region (bp)	6,448,323	86.41
DNA G + C content (bp)	4,683,660	62.76
Number of scaffolds	1	
Number of contigs	5	
Total genes	7,388	100.00
RNA genes	70	0.95
rRNA operons	1*	
Protein-coding genes	7,318	99.05
Genes with function prediction	5,982	80.97
Genes assigned to COGs	5,882	79.62
Genes assigned Pfam domains	6,121	82.85
Genes with signal peptides	654	8.85
Genes coding transmembrane proteins	1,735	23.48

*1 copy of 5S, 2 copies of 16S and 1 copy of 23S rRNA genes.

**Table 4:** **Number of protein coding genes of *Mesorhizobium loti *** **NZP2037 associated with the general COG functional categories**

**Code**	**Value**	**% age**	**COG category**
J	205	3.14	Translation, ribosomal structure and biogenesis
A	0	0.00	RNA processing and modification
K	603	9.24	Transcription
L	242	3.71	Replication, recombination and repair
B	7	0.11	Chromatin structure and dynamics
D	34	0.52	Cell cycle control, mitosis and meiosis
Y	0	0.00	Nuclear structure
V	82	1.26	Defense mechanisms
T	262	4.01	Signal transduction mechanisms
M	320	4.90	Cell wall/membrane biogenesis
N	51	0.78	Cell motility
Z	2	0.03	Cytoskeleton
W	1	0.02	Extracellular structures
U	142	2.17	Intracellular trafficking and secretion
O	197	3.02	Posttranslational modification, protein turnover, chaperones
C	381	5.84	Energy production conversion
G	624	9.56	Carbohydrate transport and metabolism
E	798	12.22	Amino acid transport metabolism
F	95	1.46	Nucleotide transport and metabolism
H	242	3.71	Coenzyme transport and metabolism
I	275	4.21	Lipid transport and metabolism
P	267	4.09	Inorganic ion transport and metabolism
Q	213	3.26	Secondary metabolite biosynthesis, transport and catabolism
R	811	12.42	General function prediction only
S	675	10.34	Function unknown
-	1,506	20.38	Not in COGS

## Conclusion

The *M. loti* NZP2037 genome consists of a single chromosome of 7.46 Mb predicted to encode 7,388 genes. The sequencing was completed to the stage where a single scaffold comprising 5 contigs was obtained. NZP2037 differs from other well-characterised *M. loti* strains in that it is able to form effective nodules on the host *L. pedunculatus (syn. L. uliginosus)*[2]. The molecular basis of this extended host range remains unknown; however NZP2307 carries additional *nod genes* (*nodU, nodFEG* and a second copy of *nodA*) not found in other well-characterised *M. loti* strains such as MAFF303099 and R7A [7]. Preliminary studies suggest it may also produce some different surface polysaccharides to R7A [11,12].

Previously it was demonstrated that NZP2037 contains a transmissible plasmid of 240 MDa (approximately 360 kb) designated pRlo22037a [25]. Strain PN4010, a plasmid-cured derivative of NZP2037, showed enhanced levels of nitrogen fixation and competitiveness on *Lotus pendunculatus* versus the wild-type. Reintroduction of the plasmid into PN4010 returned the strain to the wild-type phenotype [51]. A type IV secretion system consisting of a trb gene cluster (Locus tags 7041-7051 coordinates 70104004-7113626) and *traG* (locus tag 6995 coordinates 7068484-7070472) highly similar (80-98% amino acid identity) to that of the *M. loti* strain MAFF303099 pMlb plasmid are located at the end of the scaffold. This finding and comparison of the genome sequence with that of *M. loti* strains R7A and MAFF303099 suggests that the right end of the single large scaffold may in fact be a large plasmid.

## Competing interests

The authors declare that they have no competing interests.

## Authors’ contributions

JS and CR supplied the strain and background information for this project and contributed to the assembly of the manuscript with WR, TR supplied DNA to JGI and performed all imaging, WR coordinated the project and all other authors were involved in either sequencing the genome and/or editing the paper. All authors read and approved the final manuscript.
